# Housing as a social determinant of health and wellbeing: developing an empirically-informed realist theoretical framework

**DOI:** 10.1186/s12889-020-09224-0

**Published:** 2020-07-20

**Authors:** Steve Rolfe, Lisa Garnham, Jon Godwin, Isobel Anderson, Pete Seaman, Cam Donaldson

**Affiliations:** 1 Faculty of Social Sciences, University of Stirling, Stirling, FK9 4LA UK; 2 Glasgow Centre for Population Health, Olympia Building, Bridgeton Cross, Glasgow, G40 2QH UK; 3 School of Health and Life Sciences, Glasgow Caledonian University, Cowcaddens Road, Glasgow, G4 0BA UK; 4 Yunus Centre for Social Business and Health, Glasgow Caledonian University, Cowcaddens Road, Glasgow, G4 0BA UK

**Keywords:** Housing, Health, Social determinants, Causal mechanisms, Realist evaluation

## Abstract

**Background:**

The role of housing as a social determinant of health is well-established, but the causal pathways are poorly understood beyond the direct effects of physical housing defects. For low-income, vulnerable households there are particular challenges in creating a sense of home in a new tenancy which may have substantial effects on health and wellbeing. This study examines the role of these less tangible aspects of the housing experience for tenants in the social and private rented sectors in west central Scotland.

**Methods:**

The paper analyses quantitative data from a mixed methods, longitudinal study of tenants from three housing organisations, collected across the first year of their tenancy. The paper postulates causal hypotheses on the basis of staff interviews and then uses a Realist Research approach to test and refine these into a theoretical framework for the connections between tenants’ broader experience of housing and their health and wellbeing.

**Results:**

Housing service provision, tenants’ experience of property quality and aspects of neighbourhood are all demonstrated to be significantly correlated with measures of of health and wellbeing. Analysis of contextual factors provides additional detail within the theoretical framework, offering a basis for further empirical work.

**Conclusions:**

The findings provide an empirically-informed realist theoretical framework for causal pathways connecting less tangible aspects of the housing experience to health and wellbeing. Applying this within housing policy and practice would facilitate a focus on housing as a public health intervention, with potential for significant impacts on the lives of low-income and vulnerable tenants. The framework also offers a basis for further research to refine our understanding of housing as a social determinant of health.

## Background

Housing is often cited as an important social determinant of health, recognising the range of ways in which a lack of housing, or poor quality housing, can negatively affect health and wellbeing [1–4]. However, the causal pathways from housing to health are inherently complex, as with all the social determinants of health [5], so many of these pathways are neither fully conceptualised, nor empirically understood. This paper aims to develop an empirically-informed theoretical framework to elucidate some of the possible causal pathways between less tangible aspects of housing experience and health and wellbeing, for low-income households in rented accommodation. It is concerned with the subjective experience of housing, from the perspective of low income household in rented accommodation.

The causal relationships between tangible physical housing defects and poor health outcomes are widely accepted [6, 7], with clear evidence of negative physical health effects of toxins within the home, damp and mould, cold indoor temperatures, overcrowding and safety factors [4, 6–8], and also of negative mental health effects arising from cold indoor temperatures, overcrowding/lack of personal space, and damp and mould [4, 6, 7, 9]. Moreover, analysis of the impact of housing improvement interventions provides evidence for causal direction and pathways [10, 11].

Beyond these impacts of physical aspects of housing, the literature regarding health impacts of less tangible aspects of the housing experience is relatively sparse, although the literature around the notion of ‘home’ provides some theoretical and empirical starting points. The social, psychological and cultural value of home as something more than the material object of housing has long been recognised [12], indicating the range of ways in which dwellings offer sites of control, autonomy and socialisation, and a basis for social identity and status [13, 14]. Thus from the perspective of ‘ontological security’ [15, 16], the home is seen as providing a secure base from which people can develop confidence in self and social identity [17, 18]. Moreover, research across different housing sectors [19, 20] and examining the specific experiences of different social groups [21–23] suggests that the subjective experience of housing and housing services can be important in creating a sense of home and underpinning ontological security.

Taking this a stage further, the work of Kearns and colleagues examining the ‘psychosocial benefits of home’ [24–26], has gone some way to develop theoretical and empirical connections between housing, home and wellbeing. Their findings suggest that aspects of control, autonomy, status and empowerment are related to measures of wellbeing, with some variance in relative importance between households with different characteristics, but the use of cross-sectional data restricts the examination of causality. The evidence from longitudinal panel datasets reviewed by Clapham et al. [27] provides more evidence for the causal effect of physical housing quality and tenure on measures of subjective wellbeing, but these studies face difficulty in identifying mediators, many of which appear to relate to the psychosocial aspects of home, including autonomy, security and status.

Examining health and wellbeing impacts of aspects of housing beyond bricks and mortar is particularly important in the context of declining rates of home ownership and consequent increases in renting across much of Europe and the US, following the Global Financial Crisis [28–30]. Aside from the obvious relevance of tenure security for this growing group of tenants, the role of landlords and housing organisations may be salient, particularly in terms of the constraints that may be placed on tenants’ agency in generating their own sense of home.

This paper attempts to make progress in this area, by developing an empirically-informed, realist theoretical framework for causal pathways linking less tangible aspects of housing as experienced by tenants to health and wellbeing outcomes. Utilising quantitative data from a mixed methods, longitudinal study of tenants in Scotland, the paper employs realist analysis [31, 32] to test and refine four hypotheses relating to: relationships between tenants and their housing provider; tenants’ experience of property quality; affordability; and aspects of neighbourhood and social support. Firstly, we focus on *whether* the hypotheses are supported by the evidence. Secondly, we consider the contextual factors which play a role in determining *who is most affected and in what circumstances* [31]. To undertake this analysis, the study focuses on new tenants, conceptualising the change of tenancy and the related changes in housing experience and housing service as a complex intervention in the lives of participating tenants [33]. The paper is inherently exploratory, attempting to develop and refine a realist understanding of the causal pathways which may link less tangible aspects of the housing experience to health and wellbeing, on the basis of empirical data, to provide a framework for further analysis and research. Further analysis of the qualitative data from the study, to elaborate the precise nature of the causal mechanisms involved will be the subject of a later paper.

## Methods

This study treats the entire housing experience as the intervention from which health and wellbeing impacts result. It seeks to understand the mechanisms through which that subjective experience generates impacts, the different contexts in which those mechanisms operate and impacts vary, and why. It focuses, in particular, on the less tangible aspects of that housing experience, owing to a lack of empirical evidence in the literature. It uses realist methodology in order to achieve this. In this section we provide an introduction to realist evaluation (RE) for readers unfamiliar with this methodology, highlighting key characteristics which are of particular relevance to this study. We explain why we selected this methodology and how we employed it in practice.

RE is an established methodology within the school of theory-based evaluation (TBE) approaches. These methodologies as a whole attempt to move away from before-and-after evaluation designs, using theory to attempt to uncover and understand the causal processes and mechanisms at play within any policy or programme [34, 35].

### Understanding causality within realist evaluation

As Gates & Dyson [36] argue, there is a ‘growing acknowledgement that there are multiple ways to think about causal relationships’ and therefore a diversity of ways to make causal claims in social science. RE starts from a realist view of causality, which has two important features. Firstly, causality is conceptualised as generative rather than successionist. Generative causation emphasises that it is the latent ‘powers and liabilities’ within things which generates effects in particular contexts [37]. Whilst methodologies based on successionist causality, such as RCTs, attempt to control for contextual influences, RE deliberately incorporates context to examine how it influences the operation of causal mechanisms. Thus, the RE position is that research needs to focus not on whether programmes work in a general sense, but on ‘what works, for whom, in what circumstances’ [31], often now extended to include questions of ‘how and why?’ This is particularly valuable in situations where ‘interventions’ and contexts are interconnected in complex ways, such as social situations where the intervention is shaped by the agency of the beneficiary.

Secondly, building on the realist notion of a ‘stratified reality’ [31, 38], RE recognises that most mechanisms will be hidden. Whilst many elements of social reality, such as human behaviour or the existence and activities of institutions are directly observable, the mechanisms which generate social outcomes are often hidden within individual reasoning or complex organisational interactions and hence are not necessarily tangible. As Westhorp [37] argues, the invisible nature of mechanisms arises because they (often) operate at different levels of the system than the outcome, they operate at different timescales to the outcome, and they depend on relationships and interactions, some of which cannot be observed. However, the practical impossibility of directly observing such causal mechanisms does not preclude understanding, it merely highlights the need for theory to provide an explanation. In a sense, this perspective provides an additional angle to the truism that ‘correlation does not imply causation’, since the implication is that correlation requires a theory of mechanisms to identify the likelihood of a causal relationship.

### Understanding realist evaluation

Building on these understandings of causality and the nature of mechanisms, RE departs from experimental methodologies that attempt to control contextual influences to isolate the effect of particular interventions or mechanisms. Rather, it explicitly recognises that mechanisms operate differently in different contexts and therefore develops causal hypotheses in the form of ‘Context-Mechanism-Outcome Configurations’ (CMOCs), attempting to identify how causal mechanisms may operate to generate outcomes within particular contexts.

In practical terms, RE proceeds in a cyclical fashion to iteratively refine our understanding of mechanisms and the contexts within which they operate to generate outcomes [39]. Initial, tentative theories in the form of hypothesised CMOCs are developed from existing evidence in the literature and stakeholder engagement, employing ‘abductive reasoning’, which Jagosh et al. [40] define as “inference to the best explanation”. Mixed methods data collection is then utilised to examine the ‘outcome regularities’ which relate to these initial CMOCs, in two phases. Firstly, quantitative data is employed to examine the patterns of outcomes across different contexts in order to test and refine the initial CMOCs. The causal theories represented by these CMOCs are then refined further by using qualitative data to elucidate the underlying mechanisms through exploring and triangulating stakeholders’ understandings. These refined CMOCs provide a more nuanced picture of what works, for whom in different circumstances, which can be utilised in practice and also provides the starting point for a further iteration of the realist research process.

Importantly, RE attempts to develop theoretical understanding at different levels. On the one hand, the aim is to develop ‘middle-range theory’ (MRT) [41] regarding causal mechanisms. MRT lies between the (impossible) grand, unified theory of social behaviour, organisation and change, and the very specific understandings of particular contexts [31, 42]. On the other hand, individual RE studies aim to improve the specification of CMOCs, thereby “learning more and more about less and less” [31] in order to enhance our understanding of the particular contexts within which causal mechanisms work for particular groups of people. The process of knowledge cumulation within RE involves traversing repeatedly between abstraction and specification to refine the MRT and examine how it applies in particular contexts [31]. This paper focuses only on the first of these two elements, examining the outcome regularities exhibited within the quantitative data in order to develop a middle-range theoretical framework. The second stage of this analysis will be presented elsewhere.

### Rationale for using RE in this study

This research attempts to examine possible causal pathways between the less tangible aspects of the housing experience and health and wellbeing outcomes. More specifically, we set out to investigate these impacts by studying tenants entering a new tenancy, conceptualising this change as a complex intervention in the lives of these tenants. A number of aspects of this area of study suggested the value of using RE.

Firstly, the the intervention being studied is both multi-faceted and complex, in the sense that there are multiple, interacting components, including the agency of tenants and housing staff [43]. Where interventions as well as outcomes are emergent, and where context is likely to be important, an evaluation approach is needed which can operate at the level of the system [44]. RE explicitly attempts to grapple with the fact that society and human behaviour are in a permanent state of self-transformation, by recognising the mutating nature of social programmes and the role of agency in generative causation [32, 45]. Whilst we can attempt to understand the mechanisms which may generate health and wellbeing outcomes, it is neither possible nor practically useful to attempt to separate ‘intervention’ elements from the complex open system within which they take place. Rather, we used RE in order to examine the ‘interventional systems’ [46] within which health and wellbeing change may be generated for and by tenants.

Secondly, given that tenants are active agents within their housing experience, the notion of generative causality is likely to be valuable in exploring the pathways between aspects of housing experience and health and wellbeing outcomes. Moreover, whilst health and wellbeing can be measured at static points in time, there is a sense in which health and (especially) wellbeing are emergent properties which are constantly in flux. Hence, understanding the causal relationships is likely to require an exploration of multiple, inter-related mechanisms which operate on different timescales [37].

We therefore employed RE methodology in order to examine the complex, contingent and emergent nature of the less tangible aspects of housing. We aimed to examine the causal impact of the subjective housing experience, and to clarify the nature of the mechanisms involved and the contexts influencing their operation.

### Project design

In order to explore a range of possible mechanisms and contexts, we worked with three quite different housing organisations, described in Table 1. The organisations operate across the social and private rented sectors, but with a similar client group of low-income tenants. Low-income households are most likely to be at risk from poor health and wellbeing and more likely to experience poor housing. They therefore represent the portion of the population for whom it is more important to understand the relationship between housing and health and wellbeing, if we are to effectively address and reduce health inequalities.

**Table 1:** Outline of participant organisations

**Housing Association**	
• Community-based Housing Association, providing social rented housing and operating a subsidiary regeneration organisation which focuses on employment and community development. Aims to provide affordable housing in the social rented sector (SRS) to low income households with a variety of needs, as well as contributing to community sustainability and regeneration through non-housing activities. Owns and manages around 5500 properties.	
**Letting Agency**	
• Social enterprise letting agency which manages property for private rented sector (PRS) landlords. Combined with investment arm which purchases its own property and rents it, through the letting agency arm. Social mission to provide high quality housing in the PRS to vulnerable households. Provides tenancy support service, funded from service charge income. Manages around 250 properties on behalf of private landlords and owns a further 200.	
**Rent Deposit Schemes**	
• Voluntary sector organisation running two Rent Deposit Schemes (RDS), which facilitate access to the PRS for households at risk of homelessness. Provides deposit guarantee to enable vulnerable households without savings to access PRS tenancies, as well as a level of tenancy support over the first year of the tenancy. Tenants are expected to save up their deposit over the first year of their tenancy instead of being asked to provide it up-front, before their tenancy begins. Combined, the two schemes support around 100 people into tenancies each year.	

#### Phase 1 – developing the initial hypotheses

In the first phase of the research, individual semi-structured interviews were carried out with 23 staff across the three organisations, in order to uncover the program theories underlying their practice, with specific reference to potential health and wellbeing impacts. Interviewees were selected in order to provide a cross-section of staff, encompassing different aspects of each organisation’s approach to working with tenants. Table 2 provides an overview of the interviewees in each organisation.

**Table 2:** Overview of scoping study interviewees

Organisation	Interviewees
A Housing Association	• Assistant Director of Housing Services • Housing Manager • Housing Officer • Concierge • Regeneration Manager • Community Support/Development Officers × 2 • Cultural Officer • Development Officer (money advice service)
B Letting Agency	• Director • Assistant Director • Tenancy Support Officer • Property Inspection Officer
C Rent Deposit Schemes	• Service Manager • Team Leader × 2 • Senior Development Officer • Development Officer/Support Worker × 4 • Admin Worker × 2

The data from these interviews provided the implicit causal understandings of practitioners, which was then combined with existing evidence from the literature to examine the plausibility of the suggested mechanisms and contextual factors, and to develop the initial, tentative CMOCs, as laid out in Table 3. There were some differences between the organisations regarding the specific contextual factors that might be relevant, but across the interviews the same four mechanisms were seen as likely to have a notable impact on health and wellbeing. Given the similar conceptions of potential mechanisms across the organisations, we therefore set out to collect data from tenants which would enable us to test and refine these CMOCs, treating the housing organisation as just one contextual factor amongst many that might impact upon the tenants’ housing experience.

**Table 3:** CMO-Cs through which housing situation may affect health and wellbeing and potential contextual influences

CMO-C	Contextual factors	Mechanism	Outcome
1	• Security of tenure • Tenancy support • Responsiveness of landlord to problems • Expectations, situation and capacity of tenant	Positive tenancy experience reduces stress and provides tenants with autonomy and control	Improved health and wellbeing
2	• Level of investment in property prior to tenancy	Quality housing provides tenants with a comfortable space in which to relax and a sense of status	Improved health and wellbeing
3	• Rent levels • Income levels • Benefits system (especially changes) • Landlord responses to financial issues	Affordable housing reduces financial stress and frees up income for other expenditure	Improved health and wellbeing
4	• Community development activities of landlord • Opportunities for choice of neighbourhood • Existing networks of tenants • Tenancy support	Good neighbourhood environment and supportive social/community networks around housing location reduce stress and increase opportunities for socialisation	Improved health and wellbeing

#### Phase 2 – data collection

Data was collected from a cohort of new tenants, over the period 2016–2018. All new tenants were invited to participate in the study, being given initial information by housing organisation staff prior to a more detailed conversation and opt-in consent process with the research team. Participation was voluntary, with around 50% of new tenants agreeing to take part in the study. Data was collected through structured interviews carried out at three time points: the start of the tenancy (Wave 1), collecting background data on tenants’ prior housing situation; 2–4 months into the tenancy (Wave 2); and 9–12 months into the tenancy (Wave 3). At each wave, quantitative data was collected on satisfaction with various aspects of the housing service, community and social networks, health and wellbeing, financial circumstances and demographics. At Waves 2 and 3, these elements were also explored qualitatively through face-to-face interviews conducted in the tenant’s home, although this data is not presented here. Table 4 sets out the numbers of tenants involved at each Wave and Table 5 provides a demographic overview of the sample, based on those completing at least the first two Waves of data collection.

**Table 4:** Numbers of participating tenants at each Wave

Organisation	Wave 1	Wave 2	Wave 3
**Housing Association**	56	33	23
**Letting Agency**	50	34	17
**Rent Deposit Schemes**	15	8	5
**Total**	**121**	**75**	**45**

**Table 5:** Demographic overview of participating tenants (Wave 2 completers)

		Housing Assoc.	Letting Agency	Rent Deposit Schemes	Total
**Full sample**		**33**	**34**	**8**	**75**
Gender	Female	17	18	5	40
	Male	16	16	3	35
Age	Younger (< 35)	12	20	2	34
	Older (= > 35)	21	14	6	41
Disability	Disabled	14	5	3	22
	Non-disabled	19	29	5	53
Employment	Employed	8	23	0	31
	Not employed	25	11	8	44
Household type	Household without children	21	26	5	**52**
	Household with children	12	8	3	23
Household income	< 50% median	30	21	8	59
	50–60% median	1	4	0	5
	60–100% median	2	7	0	9
	> 100% median	0	2	0	2
Housing Benefit	Full or partial Housing Benefit	25	7	8	40
	No Housing Benefit	8	27	0	35
Previous housing situation	Social housing	9	2	2	13
	Private rented sector	8	21	2	31
	Homeless	10	5	4	19
	Other	6	6	0	12

The drop-out rates between the waves are largely due to two factors. At Wave 1, data was collected through a short telephone interview (around 15 min), whereas Waves 2 and 3 involved more onerous face-to-face interviews in the tenants’ home of around 30–60 min in length. The attrition at Wave 3 was exacerbated by the timescale of the project – some Wave 3 interviews could not be scheduled before data collection had to be completed. These patterns were relatively consistent across the three organisations and the number of tenants moving on or losing their tenancy was very small (< 5%). We also compared demographic data for the participant groups at each wave with each other and with the wider population of new tenants within each organisation. This analysis showed only minor differences, suggesting a limited degree of selection bias.

For the purposes of this analysis, the definition of health aligns with that of the World Health Organization: “a state of complete physical, mental and social wellbeing, and not merely the absence of infirmity” [47]. The definition of wellbeing itself is complex, but we use it to mean a combination of positive psychological state and a functional balance between individual resources and challenges [48]. Crucially, these conceptions of health and wellbeing overlap considerably, reflecting the growing evidence base indicating that psychological wellbeing is a significant determinant of physical health, particularly over the life course [49] and that measures of wellbeing are highly correlated with measures of health [50, 51]. As both a close analogue to and a determinant of health, we suggest that wellbeing is an important outcome to consider in housing research [27].

On the basis of these definitions and given the low likelihood of significant impacts on clinical health indicators arising from social determinants within a single year, we used three self-report questions to measure health and wellbeing at each wave. Whilst self-rated health status has clear limitations, there is good evidence to suggest that it provides a reliable indicator of objectively measured health [52]. We employed the World Health Organization’s 5-point wellbeing scale (WHO5) as an internationally-validated measure of wellbeing [51]. As a general measure of health, we used a self-rated health status question drawn from the Scottish Household Survey. Unsurprisingly, the data from this question showed no significant change between waves, although it was still useful in demonstrating that our sample was somewhat more unhealthy than the general population (17% ‘bad’ or ‘very bad’ health in the sample, compared to 9% in SHS 2017 data), as would be expected for this group of vulnerable and low-income households. A focus group and pilot interviews with tenants prior to the main data collection phase had indicated that most people were likely to interpret this question relatively narrowly as relating to physical disease, which we did not expect to be substantially affected by the housing experience within a year. Moreover, this pilot work indicated that broader conceptions of health and wellbeing overlap considerably in the public mind, reflecting the connections demonstrated in the literature. We therefore included an additional question on self-rated change in overall health and wellbeing and deliberately situated it alongside qualitative exploration to create a focus on the broader conception of health and wellbeing as a combination of physical state, mental state and functioning.

Independent variables were selected from existing questions in national surveys (Scottish Household Survey and Scottish Social Housing Charter indicators) to measure aspects of the housing experience which might plausibly trigger each of the mechanisms in Table 3. Given the RE conception of mechanisms as hidden, these variables do not attempt to measure the mechanisms directly, but to provide an indication of the potential that the theorised mechanism has been triggered, when combined with the outcome data. Additional questions relating to contextual aspects were also asked, including demographics and previous housing situations. The key outcome and independent variables are set out in Table 6. More information on these variables and their related questions available in the supplementary material.

**Table 6:** Key variables used to explore hypothesised causal pathways

Hypothesis	Variable	Type of data
Independent variables		
1	Overall satisfaction with housing organisation	5-point Likert-style scale
1	Comparison of current and previous experience of renting	5-point rating from ‘A lot better’ to ‘A lot worse’
2	Rating of property quality	5-point rating from ‘Very good’ to ‘Very poor’
2	Satisfaction with maintenance service	5-point Likert-style scale
3	Rating of ability to cope financially over the last few months	5-point rating from ‘All of the time’ to ‘Never’
3	Rating of ability to cope with paying rent over the last few months	5-point rating from ‘All of the time’ to ‘Never’
4	Rating of neighbourhood quality	4-point rating from ‘Very good’ to ‘Very poor’
4	Index created from four social support questions^a^	Index (5-point Likert-style scale for each question)
Dependent variables		
All hypotheses	World Health Organization 5-point Wellbeing Scale (WHO5) – score created from five statements of wellbeing over the preceding two weeks	6-point rating from ‘All of the time’ to ‘At no time’
All hypotheses	Self-rated change in health and wellbeing since moving into new property (self-rated H&WB change)	5-point rating from ‘A lot better’ to ‘A lot worse’

^a^ These questions were included to create an index, drawing on their use in other social surveys, such as the Scottish Household Survey. Cronbach’s alpha is > 0.75 at all three Waves

#### Phase 3 – analysis to test and refine the theories

The analysis of the quantitative data was undertaken in two stages. Firstly, the data was used to examine the outcome regularities and thereby test whether the hypothesised mechanisms appeared to be operating to generate impacts on health and wellbeing. Bivariate tests were carried out using the full sample (using Spearman’s Rho for non-parametric data) to examine correlations between the independent variables related to each hypothesis and two health and wellbeing outcome variables. Outcome regularities evidenced by correlations do not in themselves provide clear evidence of causality, but provide a basis for further investigation of the underlying mechanisms and the contextual factors which may be affecting their operation.

Importantly, the two health and wellbeing variables serve different purposes within the analysis. The WHO5 scale provides a validated, internationally-recognised measure [51], focused primarily on the positive psychological state aspect of wellbeing. Due to challenges in contacting tenants prior to their move, 42% of participants did not complete the Wave 1 WHO5 questionnaire until more than 2 weeks after their move-in date. As such, Wave 1 WHO5 does not reliably describe pre-move wellbeing for all tenants and therefore cannot be used to assess improvement pre- and post-move across the whole sample. Hence it is primarily used within the analysis to examine potential correlations within each Wave, testing for ‘static’ health and wellbeing effects of aspects of housing service, housing quality, financial coping and neighbourhood. The self-rating of health and wellbeing collected at Waves 2 and 3, provides a direct measure of tenants’ perspectives on what has changed for them since the start of their new tenancy and is therefore used within the analysis to examine potential correlations across Waves, testing for ‘change’ effects of the same aspects of housing situation. Since the question specifically asks for self-rated change since the start of the tenancy, this variable provides a direct indicator of tenants’ perceptions of the impact of their change in housing situation on their health and wellbeing.

Secondly, further tests (again using Spearman’s Rho) were conducted for sub-populations within the full sample, in order to examine potential contextual factors which may be influencing the operation of mechanisms within each hypothesised CMO-C, including demographic characteristics, socio-economic status and household type, as well as differences between the organisations. This analysis was carried out using the Wave 2 data, in order to provide a sufficient sample size at sub-population levels. All of the sub-populations defined in Table 5 above were tested in this analysis, but household type was simplified into households with children and those without, whilst the analysis based on household income categories is not presented here, as the small numbers of households outside the lowest income category makes comparison between income groups impossible. Most variables have very few missing values and analysis suggests that they are missing completely at random (with one exception highlighted in the findings), so pairwise exclusion was used. The Rent Deposit Schemes (RDS) are also excluded from the organisational breakdown analysis because the low numbers of participating tenants make it impossible to perform meaningful tests. RDS tenants are included in the other sub-population tests.

## Results

### Examining outcome regularities across full sample

**Fig. 1:**
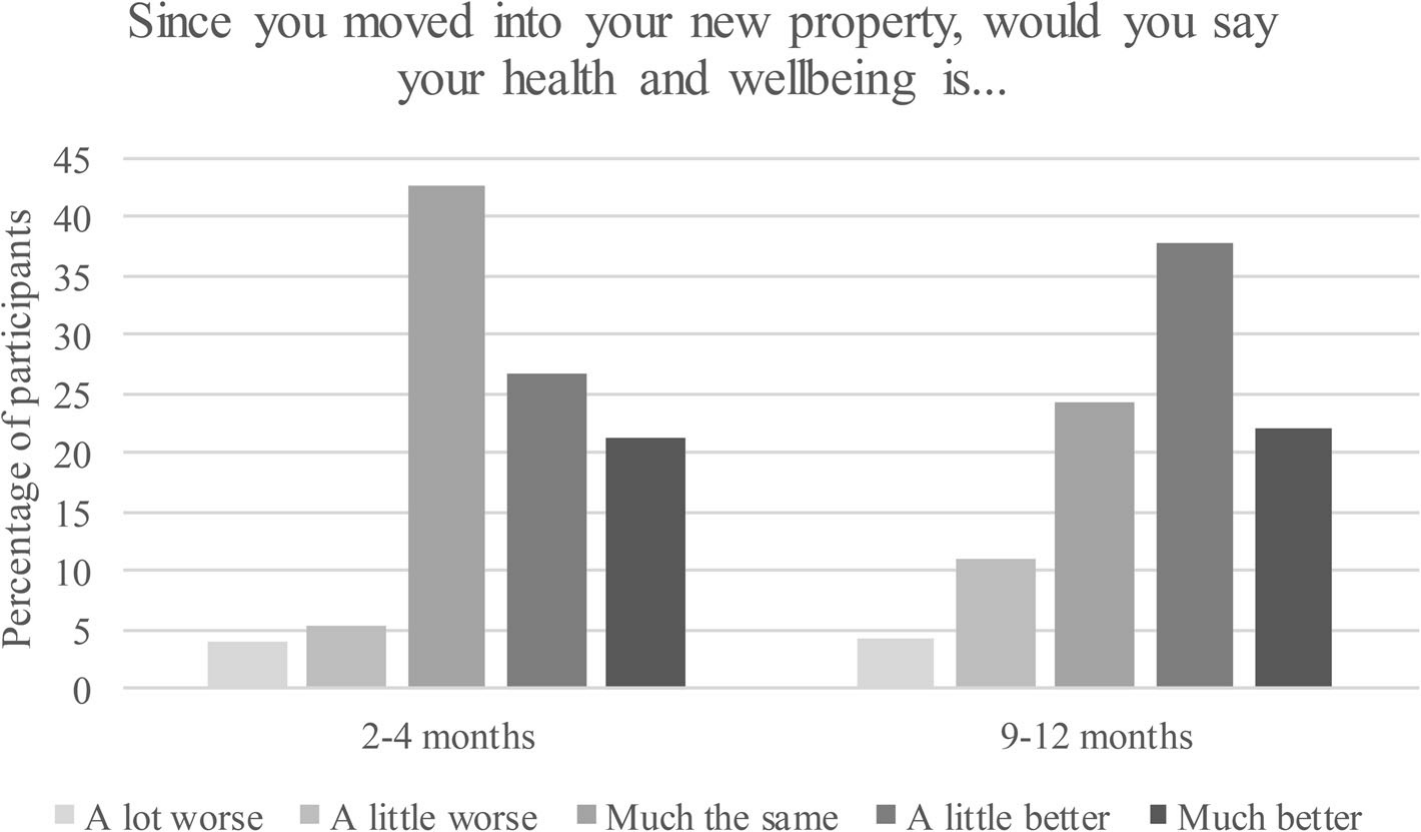
Health and wellbeing change from start of tenancy

The data for both outcome variables indicates improvement in health and wellbeing across the first year of the tenancies. Figure 1 shows the data for tenants’ self-rated health and wellbeing change at Waves 2 and 3, showing a clear improvement at both time points.

**Fig. 2:**
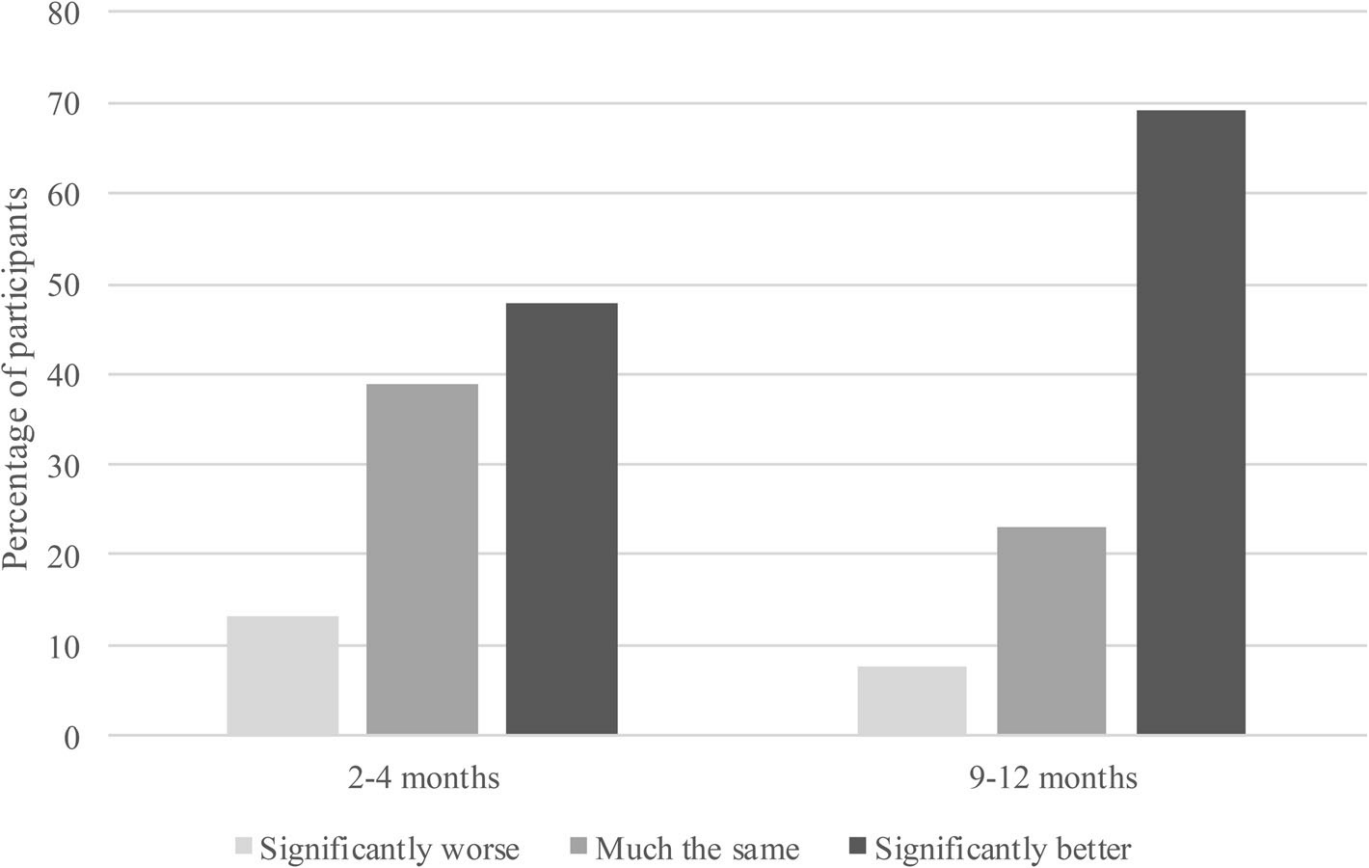
Change in WHO5 wellbeing score from start of tenancy. Note:This data relates only to those tenants who completed their Wave 1 WHO5 questionnaire prior to moving in to their new tenancy

Figure 2 provides the data for change in tenants’ WHO5 score from the start of their tenancy to the Wave 2 and 3 time points.^1^ Again, this data suggests that tenants’ wellbeing is improving over time in their new tenancy. A similar pattern can be seen for the tenants of each participant organisation when analysed separately.

This overall picture of improving health and wellbeing by comparison with tenants’ prior situations suggests that there may be aspects of their new housing experience which are generating this change. The first stage of the data analysis examines the possible role of the hypothesised CMO-Cs by testing for correlations with related independent variables. A summary of this analysis is provided in Table 7. The columns labelled ‘Wave 2′ and ‘Wave 3′ set out the results of the tests using the WHO5 index as the dependent variable, whilst those labelled ‘Wave 1–2′ and ‘Wave 1–3′ provide the results for tests using the variable for self-rated health and wellbeing change since the start of the tenancy.

**Table 7:** Summary of hypothesis tests on full sample

Hypothesis		Independent variable	Wave 2 (WHO5)		Wave 3 (WHO5)		Wave 1–2 (HWB change)		Wave 1–3 (HWB change)	
			Rho	Sig.	Rho	Sig.	Rho	Sig.	Rho	Sig.
1	Experience of secure, stable tenancy reduces stress and provides tenants with a secure base from which to exercise autonomy	Overall satisfaction with housing organisation	0.27	0.02*	0.28	0.06	0.43	0.001***	0.26	0.09
		Comparison of current and previous experience of renting^a^	NA	NA	NA	NA	0.38	0.002**	0.23	0.1
2	Quality housing provides tenants which a comfortable space in which to relax and a sense of status	Rating of property quality	0.28	0.02*	0.30	0.05*	0.31	0.007**	0.29	0.05*
		Satisfaction with maintenance service	0.18	0.2	0.095	0.6	0.46	0.009**	−0.033	0.9
3	Affordable housing reduces financial stress and frees up income for other expenditure	Rating of ability to cope financially over the last few months	0.32	0.005**	0.57	0.001***	0.14	0.2	0.18	0.2
		Rating of ability to cope with paying rent over the last few months	0.047	0.7	0.081	0.6	0.030	0.8	0.17	0.3
4	Good neighbourhood environment and supportive social/community networks around housing location reduce stress and increased opportunities for socialisation	Rating of neighbourhood quality	0.46	0.001***	0.44	0.003**	0.25	0.04*	0.20	0.2
		Index created from four social support questions	0.33	0.005**	0.30	0.04*	0.28	0.02*	−0.001	1.0

*p < 0.05, **p < 0.01, ***p < 0.001. N = 75 at Wave 2, N = 45 at Wave 3

^a^This variable is explicitly about change in rental experience and therefore was not tested against the ‘static’ WHO5 measure

These results provide patterns of ‘outcome regularities’ in the language of RE, which suggest a number of potential refinements to the CMOCs. For each hypothesised causal pathway, the presence or absence of significant correlations provides evidence as to whether the relevant mechanism may be operating, bearing in mind that the subsequent stage of the analysis may qualify these findings by considering the contextual factors involved.

Firstly, in terms of the experience of the property and housing service, there appears to be some support for Hypotheses 1 and 2. All the variables relating to these aspects of housing experience, with the exception of maintenance service satisfaction at Wave 2, show a significant correlation with health and wellbeing outcomes. In particular, the strength of the correlations in relation to change in health and wellbeing from Wave 1 to Wave 2 suggests the possibility of a causal connection which merits further investigation.

Secondly, the data relating to tenants’ self-rated financial coping shows that whilst overall financial coping is strongly correlated with wellbeing at Wave 2, this effect does not appear in between-wave analyses. Alongside this, there is no significant correlation between tenants’ self-rated ability to cope with paying their rent and their health and wellbeing, either within or across the Waves. This combination suggests that Hypothesis 3 is not supported by this data, since neither rent nor a change in housing situation affects health and wellbeing as measured in this study.

Thirdly, in terms of neighbourhood and social support, there is support for Hypothesis 4, since there are significant correlations between neighbourhood quality and the social support index, and health and wellbeing.

Whilst correlation does not imply causation, these patterns in the data provide indications of potential relationships which merit further investigation in order to understand whether causal mechanisms are involved and, if so, which contextual factors influence their operation.

Clearly care must also be taken in drawing conclusions about differences in correlations between the ‘static’ and ‘change’ tests, since the dependent variables measure somewhat different aspects of health and wellbeing. Nevertheless, variations in the significance level of the correlations across hypotheses suggests that it is the experience of the housing and the housing service *relative to a tenant’s previous experiences,* and not the current experience of the housing or service in itself, that may predict improvements in health and wellbeing. Conversely, the effects of neighbourhood quality and the availability of local social support appear to show the opposite pattern, in that current experience may predict changes in health and wellbeing outcomes, regardless of tenants’ previous housing situation(s).

The overall pattern of correlations at Wave 3 is very similar to that at Wave 2, although there appears to be some drop-off in terms of the strength of the relationships. This may be due to the smaller sample size at Wave 3 (*N* = 45), which inevitably limits the strength of possible correlations by comparison with Wave 2 (*N* = 75). Furthermore, given these relatively small sample sizes, there is a possibility that selection effects may be influencing the apparent differences between Wave 2 and Wave 3. To examine this possibility, the analysis was repeated across the two waves using data from only those tenants who had completed all three Waves. This analysis showed a very similar pattern of within-Wave correlations at Wave 2 to the analysis on the full sample. Moreover, tests for difference between the group of tenants who dropped out after Wave 2 and those who continued to Wave 3 on the independent and dependent variables, as well as demographic characteristics, show no significant difference between these two groups except for one – rating of neighbourhood quality at Wave 2 (2p = 0.04). Hence selection bias does not appear to affect the analysis outlined above to any great degree. Alongside this, the between-wave analysis in Table 7 appears to show an ‘adaptation effect’, whereby the impact of housing changes on health and wellbeing diminishes over time. However, the sub-sample analysis suggests that this apparent ‘adaptation effect’ may simply be an artefact of the smaller sample size at Wave 3. Within the sub-sample there are very limited changes in significance of correlations, suggesting that the data does not demonstrate an adaptation effect.

### Exploring contextual factors

In order to explore the potential role of contextual factors in influencing the operation of the hypothesised mechanisms, the same bivariate tests were carried out on sub-populations, in order to identify potential differences in impact based on tenant characteristics and housing organisation. The analysis focuses on the change between Wave 1 and Wave 2, specifically to examine the short-term difference that a new tenancy and home with a new housing organisation makes to tenants’ health and wellbeing. Thus, the tests look for correlations between self-rated changes in health and wellbeing since the start of the tenancy and self-rated changes in various aspects of the housing experience. The outcome variable for all of the subsequent tables is therefore self-rated change in health and wellbeing. The one exception to this is overall satisfaction with the housing organisation, since this variable is not available at Wave 1. Many tenants did not have a ‘housing organisation’ at Wave 1, because they were either homeless or living with friends or family. Hence, for this test the ‘static’ variable of housing satisfaction at Wave 2 was used.

#### Hypothesis 1 – positive tenancy experience

Table 8 summarises the analysis by sub-population for the two key variables relating to overall tenancy experience: satisfaction with the housing organisation, and overall rental experience compared to previous experiences.

**Table 8:** Sub-population analysis related to Hypothesis 1

			Correlation with Wave 1–2 health and wellbeing change			
			Satisfaction with organisation		Renting experience (comparison to previous)	
		n	Rho	Sig.	Rho	Sig.
Total	Total	75	0.43***	0.001	0.38**	0.002
Organisation^a^	Housing Assoc.	33	0.51**	0.003	0.50*	0.01
	Letting Agency	34	0.38*	0.03	0.45*	0.01
Gender	Female	40	0.49**	0.001	0.33	0.05
	Male	35	0.35*	0.04	0.42*	0.03
Age	Young	34	0.55**	0.001	0.35	0.08
	Old	41	0.34*	0.03	0.41*	0.01
Disability	Disabled	22	0.51*	0.02	0.38	0.09
	Not disabled	53	0.38**	0.005	0.43**	0.005
Employment	Employed	31	0.56**	0.001	0.47*	0.02
	Not employed	44	0.35*	0.02	0.35*	0.03
Housing Benefit	Full or partial HB	40	0.46**	0.003	0.42*	0.01
	No HB	35	0.40*	0.02	0.37	0.06
Household Type	Children	23	0.42*	0.05	0.30	0.2
	No children	52	0.46**	0.001	0.42**	0.005
Previous housing situation	Homeless	19	0.54*	0.02	0.70**	0.005
	Social housing	13	0.63*	0.02	−0.18	0.6
	PRS	31	0.43*	0.02	0.38*	0.04
	Other	12	−0.053	0.9	0.58	0.2

^a^ As noted in the methodology, the RDS tenants are excluded from the analysis by organisation (in this and subsequent tables), as the numbers of participating tenants are too small to perform meaningful tests

The key finding here is that the strongly significant correlations for the whole population of participants are largely reflected in the vast majority of sub-populations, for both variables. Whilst there are differences in the level of significance between sub-populations and some sub-populations which do not show a significant correlation on the overall renting experience variable, the differences in *p* values are small and may plausibly be explained by the smaller size of some of the sub-samples.^2^ Hence tenancy experience, at least insofar as it is captured by satisfaction with the housing organisation and comparison with previous renting experiences, has a universal relationship with health and wellbeing across the different sub-populations of tenants in this study.

#### Hypothesis 2 – property quality

Table 9 summarises the analysis by sub-population for the two key variables relating to property quality: tenant rating of the overall condition of the property and satisfaction with the maintenance service.

**Table 9:** Sub-population analysis related to Hypothesis 2

		Correlation with Wave 1–2 health and wellbeing change					
		Tenant rating of property quality			Satisfaction with maintenance service		
		n	Rho	Sig.	n	Rho	Sig.
Total	Total	73	0.31**	0.007	32	0.46**	0.009
Organisation	Housing Assoc.	31	0.081	0.7	12	0.67*	0.02
	Letting Agency	33	0.56**	0.001	15	0.50	0.06
Gender	Female	38	0.44**	0.006	20	0.52*	0.02
	Male	35	0.16	0.4	12	0.33	0.3
Age	Young	33	0.50**	0.003	12	0.19	0.6
	Old	40	0.16	0.3	20	0.54*	0.01
Disability	Disabled	22	0.056	0.8	11	0.51	0.1
	Not disabled	51	0.57***	0.001	21	0.45*	0.04
Employment	Employed	31	0.46**	0.009	12	0.41	0.2
	Not employed	42	0.25	0.1	20	0.42	0.06
Housing Benefit	Full or partial HB	38	0.17	0.3	19	0.50*	0.03
	No HB	35	0.51**	0.002	13	0.35	0.2
Household Type	Children	23	0.25	0.3	11	0.086	0.8
	No children	50	0.34*	0.01	21	0.59**	0.005
Previous housing situation	Homeless	17	0.32	0.2	4	0.33	0.7
	Social housing	13	0.18	0.6	7	0.35	0.4
	PRS	31	0.51**	0.003	21	0.49*	0.02
	Other	12	0.45	0.1	0	NA	NA

Looking firstly at property quality, there are notable differences between sub-populations in terms of correlations between changes in property quality and changes in health and wellbeing. The data suggests that participants who are tenants of the Letting Agency, female, young, non-disabled, employed, not receiving Housing Benefit, with no children in the household and coming from the PRS are more likely to exhibit a correlation between change in property quality and change in their health and wellbeing. Some of these may be interconnected, inasmuch as the sub-populations are connected. For example, Letting Agency tenants are more likely to be employed, non-disabled and coming from the PRS than others in the sample. However, other characteristics, such as gender, are evenly distributed across the organisations.

Perhaps most interestingly, the data on rating of property quality at Wave 2 relative to Wave 1 does not show a significant difference between the organisations (*p* = 0.44 using Mann-Whitney U test), so it does not appear that these patterns are an artefact of differences in property standards between the housing providers in this study. That is, it appears that tenants of the Letting Agency are not more (or less) likely to be satisfied by the quality of their properties, but a change in the quality of their property is more likely to be accompanied by a change in their health and wellbeing. Further qualitative analysis is required to understand whether there is a causal link here and why this might be the case.

The patterns relating to satisfaction with maintenance are somewhat different, but again exhibit notable differences between sub-populations. Thus, participants who are tenants of the Housing Association, female, older, non-disabled, in receipt of Housing Benefit, with no children and coming from the PRS are more likely to show a correlation between change in their maintenance service satisfaction and change in their health and wellbeing. Again, some of these are likely to be related, thanks to overlaps between the sub-populations. It should be noted, however, that the data for this variable is more limited, since some tenants (e.g. those coming from the family home) did not have a maintenance service to rate at Wave 1 and others had no experience of the maintenance service in their new tenancy by Wave 2. Missing value analysis suggests that this particular variable may be somewhat biased as a result. Moreover, it could be argued that this variable is less closely related to this hypothesis than tenants’ rating of property quality, since the experience of maintenance services could also be connected to Hypothesis 1 as an element of the overall tenancy experience. Thus, any conclusions relating to the maintenance variable need to be particularly tentative.

#### Hypothesis 3 – affordability

As outlined earlier, the tests for the full sample show no significant correlations between changes in health and wellbeing and changes in either self-rated ability to cope with paying rent or to cope financially. Looking at the descriptive data for these variables, the lack of relationship between health and wellbeing and rent coping is perhaps unsurprising, given that more than 70% of participants show no change in their ability to cope with paying their rent. This likely reflects the number of tenants receiving full Housing Benefit, with 39% of the sample having their rent entirely covered.

The lack of correlation with change in self-rated health and wellbeing for the whole sample is mirrored in the sub-populations, with no correlations for change in rent coping for any sub-population except households with children (at the 5% level), and only the groups of employed or non-HB recipients showing a correlation with financial coping (also at the 5% level), as shown in Table 10. This analysis therefore suggests that the overall picture of no significant relation between change in rent or financial coping and health and wellbeing is also present across the various sub-populations, with only very minor indications of variation between groups.

**Table 10:** Sub-population analysis related to Hypothesis 3^a^

		Correlation with Wave 1–2 health and wellbeing change					
		Tenant rating of ability to cope with paying rent			Tenant rating of ability to cope financially		
		n	Rho	Sig.	n	Rho	Sig.
Total	Total	55	0.030	0.8	75	0.14	0.2
Organisation	Housing Assoc.	23	−0.13	0.6	32	0.19	0.3
	Letting Agency	24	0.24	0.3	34	0.26	0.1
Gender	Female	29	0.31	0.1	40	0.095	0.6
	Male	26	−0.37	0.06	35	0.17	0.3
Age	Young	21	0.057	0.8	34	0.089	0.6
	Old	34	−0.054	0.8	41	0.16	0.3
Disability	Disabled	21	−0.065	0.8	22	0.12	0.6
	Not disabled	34	0.065	0.7	53	0.22	0.1
Employment	Employed	22	0.22	0.3	31	0.38	0.03*
	Not employed	33	−0.15	0.4	44	0.018	0.9
Housing Benefit	Full or partial HB	31	0.10	0.6	40	0.086	0.6
	No HB	24	−0.15	0.5	35	0.37	0.03*
Household Type	Children	17	0.51	0.04*	23	0.33	0.1
	No children	38	−0.21	0.21	52	0.074	0.6
Previous housing situation	Homeless	8	−0.23	0.6	19	0.21	0.4
	Social housing	13	0.17	0.6	13	0.47	0.1
	PRS	31	−0.22	0.2	31	−0.22	0.2
	Other	3	NA	NA	12	0.48	0.1

^a^ Note that sample sizes for rent coping are smaller, due to the number of participants who did not answer this question at Wave 1, either because they were homeless, or living with family

#### Hypothesis 4 – Neighbourhood and support networks

Table 11 summarises the analysis for the two key variables relating to neighbourhood quality and social support networks: tenant rating of the neighbourhood as a place to live and the index of social support indicators.

**Table 11:** Sub-population analysis related to Hypothesis 4

			Correlation with Wave 1–2 health and wellbeing change			
			Tenant rating of neighbourhood quality		Social support network index	
		n	Rho	Sig.	Rho	Sig.
Total	Total	75	0.25*	0.04	0.28*	0.02
Organisation	Housing Assoc.	33	0.25	0.2	0.42*	0.02
	Letting Agency	34	0.34*	0.05	0.20	0.3
Gender	Female	40	0.16	0.3	0.30	0.07
	Male	35	0.31	0.08	0.26	0.1
Age	Young	34	0.26	0.1	0.32	0.07
	Old	41	0.22	0.2	0.22	0.2
Disability	Disabled	22	0.32	0.2	0.45*	0.04
	Not disabled	53	0.26	0.07	0.19	0.2
Employment	Employed	31	0.39*	0.03	0.23	0.2
	Not employed	44	0.19	0.2	0.32*	0.04
Housing Benefit	Full or partial HB	40	0.20	0.2	0.26	0.1
	No HB	35	0.36*	0.04	0.23	0.2
Household Type	Children	23	0.061	0.8	0.17	0.4
	No children	52	0.32*	0.03	0.31*	0.03
Previous housing situation	Homeless	19	0.56*	0.02	0.30	0.2
	Social housing	13	0.15	0.6	0.36	0.3
	PRS	31	0.28	0.1	0.38*	0.04
	Other	12	0.40	0.2	0.074	0.8

Whilst both variables are significantly correlated with a change in self-rated health and wellbeing change for the sample as a whole, the sub-population analysis reveals some differences. The correlations with neighbourhood quality are significant for Letting Agency tenants, whereas those with social support are significant for Housing Association tenants. These in turn seem to be reflected by the correlations in sub-populations split by disability, employment and Housing Benefit receipt, all of which are distributed unevenly across the organisations, as shown in Table 5. Perhaps more interestingly, there are also differences between the sub-populations coming from different prior housing situations, which do not appear to reflect the differences between the organisations. Previously homeless tenants (who are more likely to be Housing Association tenants) show a significant correlation with neighbourhood quality whilst tenants coming from the PRS (who are more likely to be Letting Agency tenants) show a significant correlation with social support. For both variables there is a significant correlation for households without children, but not for those with children, which is perhaps counter-intuitive. It is somewhat difficult to hypothesise underlying reasons for these patterns of correlations from the quantitative data alone.

However, this analysis may be somewhat advanced by turning to more objective measures of neighbourhood quality. Data on the deprivation level of the areas that tenants have moved from (at Wave 1) and to (at Wave 2/3) shows that tenants’ rating of neighbourhood quality is not significantly correlated with the neighbourhood’s Scottish Index of Multiple Deprivation (SIMD) decile (2p = 0.6 using Spearman’s Rho). There is, however, a strongly significant correlation (2p = 0.001) between the Wave 1–2 change in tenants’ rating of neighbourhood quality and the change in SIMD decile. That is, tenants moving between areas with different levels of deprivation are more likely to describe a significant change in the quality of their neighbourhood than those who are moving between deprived areas. Thus, rating of neighbourhood quality appears to be a relative concept for tenants, likely based on a complex mixture of their neighbourhood history and expectations. In this context, it is worth noting the relatively limited degree of neighbourhood choice available to tenants in this study, given their predominantly low incomes. At Wave 1, 59% of tenants in this study were living in the most deprived SIMD quintile, whilst at Wave 2, 81% of tenants were in the most deprived quintile.

Picking apart these relationships between neighbourhood, social support and health and wellbeing clearly requires further investigation, since the patterns in the quantitative data are difficult to make sense of alone. Moreover, it seems plausible to suggest that these variables are particularly limited in terms of capturing the underlying mechanisms, since there are so many aspects of neighbourhood and social support which may be important for tenants, particularly given the probable interaction with previous housing experiences.

## Discussion

This analysis demonstrates that the health and wellbeing of participants in this study does appear to be affected by the change in subjective housing experience resulting from a move into a new tenancy. Moreover, where their new housing experience is positive, they are likely to describe improvements in health and wellbeing that are sustained, or even increased, over the first year. This evidence casts some doubt on notion of adaptation in the relationship between housing and wellbeing [27], although we acknowledge that longer-term data may show a different pattern.

Our analysis presents statistically significant relationships between a variety of aspects of the housing experience from tenants’ perspectives, and health and wellbeing outcomes. These enable us to identify which causal pathways are likely to be important in shaping health and wellbeing for different groups of low-income tenants in different contexts. By refining the CMO-Cs in this way, the analysis delineates a theoretical framework for ways in which less tangible aspects of housing experience can act as a social determinant of health and wellbeing. Whilst recognising that correlation is not causation and the nature of the independent variables only enables relatively broad refinements to the hypotheses, this additional specification of the causal pathways provides a strong basis for further research, particularly using qualitative data to examine the causal mechanisms involved.

### Hypothesis 1 – positive tenancy experience

This analysis suggests that tenants’ perceptions of the quality of service received from their housing provider may be an important determinant of health and wellbeing, supporting the hypothesis from the original ToC work that a version of CMO-C 1 is in operation. The results suggest that this is partly about the current service and partly about comparison with previous rental experiences, although there may be other factors which are not represented adequately by the available independent variables. This chimes with the findings from [26] that housing service satisfaction, as part of what they term ‘empowerment’, is correlated with wellbeing.

Importantly, this relationship appears to be near universal, showing significant correlations across tenants with different characteristics and backgrounds, suggesting that a positive renting experience, underpinned by a high-quality service, may be important for all tenants. It seems plausible to suggest that there may be a causal relationship here, possibly operating through mechanisms related to the sense of home that tenants can develop in a secure, stable tenancy with a housing organisation they trust to provide good service. When tenants feel that they are being treated well by their housing organisation and that their overall experience is better than previous situations, it is plausible that this will help to underpin their sense of control, autonomy and safety, with positive impacts on their wellbeing [13, 24].

Whilst the importance of positive tenancy experience across all groups of tenants is perhaps unsurprising, this CMO-C also appears to be largely unaffected by the housing sector/organisation, suggesting that formal security of tenure may be less important as a contextual factor than might be expected, at least within the context of these organisations. Importantly, this extends the existing debate regarding the links between tenure, ontological security and wellbeing which has largely focused on the distinction between ownership and renting [17, 18, 24, 25, 27]. Just as more recent analyses have suggested that other aspects of security (e.g. financial) may be more important than ownership, so these findings suggest that aspects of the tenancy experience may be more important than the legal status of the tenancy^3^ itself in some contexts, enabling tenants to feel secure and at home, with implications for health and wellbeing.

Table 12 below summarises these findings, illustrating how CMO-C 1 has been refined on the basis of this analysis.

**Table 12:** Refinement of CMO-C 1

Version	Contextual factors	Mechanism	Outcome
Original	• Security of tenure • Tenancy support • Responsiveness of landlord to problems • Expectations, situation and capacity of tenant	Positive tenancy experience reduces stress and provides tenants with autonomy and control	Improved health and wellbeing
Refined	• Previous experience and expectations of housing service • Standard of housing service (possibly including support and responsiveness)	Experience of (comparatively) good housing service reduces stress and enables tenants to gain benefits from housing as home	Improved health and wellbeing

### Hypothesis 2 – property quality

The analysis suggests that the tenant experience of property quality may also be an important determinant of health and wellbeing, supporting the hypothesis that a version of CMO-C 2 is in operation. The existing evidence base demonstrates that physical housing quality is a determinant of health where there are negative factors, such as damp or cold, that directly damage health [4, 6, 7]. It may be the case that some of these issues are relevant within our sample, although there was little evidence of such issues during the face-to-face interviews at Waves 2 and 3 (to be presented in a later paper). Indeed, the variations between sub-populations suggest that there may be different mechanisms involved which relate to other aspects of property quality beyond the basic fabric of the building. Table 13 summarises these findings, illustrating how CMO-C 2 has been refined.

**Table 13:** Refinement of CMO-C 2

Version	Contextual factors	Mechanism	Outcome
Original	• Level of investment in property prior to tenancy	Quality housing provides tenants with a comfortable space in which to relax and a sense of status	Improved health and wellbeing
Refined	• Tenant characteristics (including gender, age, disability, socio-economic status, household type – these may relate to capacity) • Previous housing experience • Property quality	Quality housing provides tenants with a comfortable space in which to relax and a sense of status	Improved health and wellbeing

There is one particular aspect of the analysis of participant sub-populations for this pathway that merits further investigation. There is a significant correlation between tenants’ rating of property quality and health and wellbeing amongst Letting Agency tenants, but not Housing Association tenants, despite overall ratings of property quality between the two organisations not being significantly different (Pearson Chi-Square 2p = 0.3). Given the notion that home is a phenomenon of individual experience [12] it seems plausible to suggest that tenants’ previous experiences and personal preferences are likely to be important in shaping their reaction to different aspects of property quality. It is possible that tenants’ previous experiences and expectations vary systematically between tenants moving into to a Housing Association property and those moving into a Letting Agency property. These possibilities clearly need further examination.

Whilst the analysis for this hypothesis included tenants’ rating of maintenance services, there is a reasonable argument to suggest that this variable could fit equally well with Hypothesis 1, being at least as much about service as property quality. Indeed, there are strong correlations across the four housing variables, which suggest that these two hypotheses are closely related in tenants’ real world experiences.

### Hypothesis 3 – affordability

Participants’ health and wellbeing is clearly correlated with their financial situation, as would be expected given the crucial role of income as a social determinant of health [2, 3]. However, these findings suggest that, for tenants in this study, there is a limited impact on health and wellbeing arising from rent levels. This is likely due to the particular housing market context in which this study was located, whereby the majority of rents were within Local Housing Allowance^4^ rates and, therefore, were either covered by Housing Benefit (for those on very low incomes) or were affordable to those in work. Amongst the three-fifths of participants who did not receive full Housing Benefit, 80% indicated that they could cope with paying their rent ‘all of the time’. Hence CMO-C 3 does not seem to be operating, at least in relation to rent payments, in this context. The hypothesis is therefore not presented here, since the lack of variation in this central element of affordable housing does not facilitate testing or refinement of the CMO-C, even though it may operate in different housing market contexts.

Nevertheless, the evidence regarding (admittedly, small) changes in tenants’ self-rated financial coping between Waves 1 and 2 suggests that there may be other aspects of finance around tenancy transitions which merit further exploration. Indeed, the strength of the correlations between overall financial coping and the WHO5 wellbeing scores suggests that any impact of housing on tenants’ financial situation has the potential to generate significant changes in wellbeing. Again, further exploration is required of precisely which aspects of tenants’ financial lives, particularly around moving home, underlie this impact on health and wellbeing.

### Hypothesis 4 – Neighbourhood and support networks

Our analysis suggests that both neighbourhood quality and social support networks may be important determinants of health and wellbeing, as hypothesised in CMO-C 4. However, there appear to be notable differences in terms of which tenants exhibit significant correlations between health and wellbeing and each of these two aspects of the wider environment around housing. Understanding the needs and aspirations of different groups of tenants, including how these might align with demographic and other characteristics, are important in further analysis of the qualitative data from this study. This is particularly the case given the markedly different opportunities that Letting Agents and Community-Based Housing Associations have to enable choice of area for tenants, or to make changes to the area in which their properties are located. Table 14 summarises these findings, illustrating how CMO-C 4 has been refined.

**Table 14:** Refinement of CMO-C 4

Version	Contextual factors	Mechanism	Outcome
Original	• Community development activities of landlord • Opportunities for choice of neighbourhood • Existing networks of tenants • Tenancy support	Good neighbourhood environment and supportive social/community networks around housing location reduce stress and increase opportunities for socialisation	Improved health and wellbeing
Refined	• Tenant characteristics (including disability, socio-economic status and household type) • Previous experience and expectations of neighbourhood/community • Aspects of neighbourhood quality (which may relate to activities of landlord and/or choice of neighbourhood) • Access to social support networks (which may relate to all of the above)	Good neighbourhood environment and supportive social/community networks around housing location reduce stress and increase opportunities for socialisation	Improved health and wellbeing

The complex patterns revealed in this analysis and the number of factors which potentially underlie the independent variables relating to neighbourhood quality and social support suggest that more evidence is required to draw firm conclusions regarding this possible causal pathway. Indeed, it would be reasonable to suggest that CMO-C 4 might be more accurately conceptualised as at least two separate pathways, since there appears to be something of a divergence in the data patterns related to neighbourhood quality and social support between different groups of tenants. Hence, whilst the experience of a good service from a housing provider may be important for all tenants to gain health and wellbeing benefits, other aspects of the housing experience and its connection to a sense of home, such as the connection between the dwelling and personal relationships [13] may be more varied between individuals.

### Strengths and limitations

The key strength of this research lies in the longitudinal approach, offering an insight into change within individual respondents and the causal dynamics at play. Combining the longitudinal aspect of the study with a realist approach has enabled us to develop a nuanced picture of the health and wellbeing impacts of less tangible aspects of the housing experience.

Limitations of the study include the relatively small sample size and the possibility that the longitudinal approach may have excluded those tenants with less stable housing pathways, although comparison of the participant sample with the wider tenant group suggests minimal difference and therefore limited selection bias. A larger sample size at Wave 3 would also have helped to explore whether the patterns visible at Wave 2 continue over a longer timescale. Further study with a larger group of tenants would be of value in exploring these issues further.

The use of self-rated measures of health and wellbeing could also be seen as a limitation, although using more objective measures of health would likely require a longer timescale, potentially exacerbating participant retention issues. The research was also conducted in one geographical area, so the findings need to be interpreted in the context of the specific housing policy and market context of west central Scotland. Moreover, whilst this study deliberately utilised general outcome measures to capture impact, further research would be of value using a range of more specific outcomes measures to examine differential effects within the broad concept of health and wellbeing.

## Conclusion

**Fig. 3:**
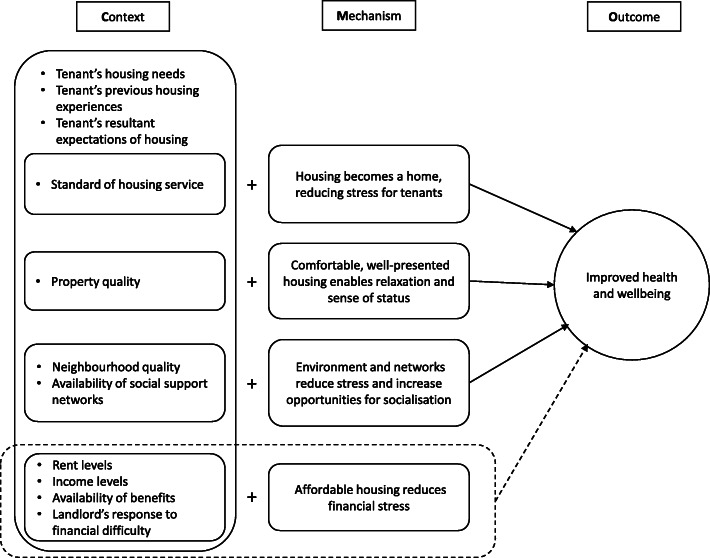
Summary of refined CMO-Cs. Note: Dotted line indicates that this CMOC is not evidenced here, but may be applicable in other housing markets

The basic human need for a home that provides more than simply shelter from the elements [12] underpins the need to understand the relationships between housing, health and wellbeing in ways that go beyond obvious problems such as damp and cold. Our analysis provides an important addition to the theoretical understanding of at least three potential causal pathways through which housing may affect health and wellbeing. Firstly, a positive tenancy experience, shaped at least in part by relationships with the housing provider is strongly correlated with health and wellbeing for all tenants, regardless of demographic characteristics or background. Secondly, aspects of the tenant experience of housing quality in addition to the basics of weatherproofing seem to be important for some tenants, in ways that are likely to be influenced by previous housing experience and current expectations. Thirdly, elements of neighbourhood quality and social support in the local area may have impacts on health and wellbeing, although with considerable variation between different groups of tenants. It may also be the case that affordability has an effect on health and wellbeing, but interestingly it appears to be relatively marginal within the particular housing market context for this study. Whilst some caution needs to be exercised in interpreting these refined CMO-Cs, given the relatively small sample size on which they are based, the longitudinal nature of the data does provide a significant insight into the patterns of change over the first year of participants’ tenancies and the potential causes for the notable improvements in health and wellbeing. The refined CMO-Cs are summarised in Fig. 3. The original CMO-C relating to affordability is included with dotted lines, to indicate its potential applicability in other housing markets.

Taken together, these CMO-Cs offer an empirically-informed realist theoretical framework for causal pathways connecting housing to health and wellbeing. This framework provides a lens through which to examine and potentially improve practice within housing organisations and housing policy, highlighting the ways in which aspects of housing service can operate as a public health intervention in the lives of tenants. Moreover, the framework offers a basis for more research to further refine and test these causal pathways. As Pawson [31, 32, 53] has consistently argued, realist evaluation and research needs to operate in a cyclical fashion, continually examining and improving theoretical models on the basis of empirical evidence to enrich our understanding of causal mechanisms, rather than developing spurious generalisations [54]. Analysis of the qualitative data from this study (forthcoming) will help to delineate the mechanisms within the framework more accurately and to examine the contextual factors in more detail, whilst other studies may also explore the role of these causal pathways in different contexts.

## Supplementary information

**Additional file 1.** Data collection instruments.

## Data Availability

The datasets generated and analysed during the current study are available in the University of Stirling’s DataSTORRE repository, http://hdl.handle.net/11667/142

## Abbreviations

CMO-C: Context-Mechanism-Outcome configuration
PRS: Private Rented Sector
RDS: Rent Deposit Scheme(s)
RE: Realist Evaluation

## Footnotes

1 ‘Significant’ change in the WHO-5 scale is considered to be 10 points when the raw score is scaled to create an index of 0–100 (Topp et al., 2015).

2 Additional chi-square tests were conducted to check against these correlations. Again, the results need to be interpreted tentatively given the sample size, but these tests suggest a similar pattern.

3 Note that the research took place before the introduction of the new Private Residential Tenancy in Scotland, which removes the time limit on PRS tenancies.

4 Local Housing Allowance is the name given to Housing Benefit for tenants in the PRS. It is limited to the 30th percentile of local rents.
