# Supplementary material for: Housing as a social determinant of health and wellbeing: developing an empirically-informed realist theoretical framework
Source: BMC Public Health. 2020 Jul 20;20:1138. doi: 10.1186/s12889-020-09224-0 (PMC7370492; doi:10.1186/s12889-020-09224-0)
Supplement: Supplementary file 1 — Additional file 1. Data collection instruments. [file 12889_2020_9224_MOESM1_ESM.docx]

**Housing as a social determinant of health and wellbeing: Developing an empirically-informed realist theoretical framework**

**Supplementary file – Data collection instruments**

Health and wellbeing outcome variables

*WHO5 wellbeing scale*

How much of the time have the following statements been true for you over the past two weeks?

- I have felt cheerful and in good spirits
- I have felt calm and relaxed
- I have felt active and vigorous
- I woke up feeling fresh and rested
- My daily life has been filled with things that interest me

Options: All of the time, Most of the time, More than half of the time, Less than half of the time, Some of the time, At no time, Don’t know

Responses are coded 5-0, summed and multiplied by 4 to create an index from 100 to 0.

*Self-rated health status (Source: Scottish Household Survey)*

How is your health in general?

Options: Very good, Good, Fair, Bad, Very bad, Don’t know

*Self-rated change in health and wellbeing*

Since you moved into your new property, would you say your health and wellbeing is…

Options: Much better, A little better, Same as before, A little worse, A lot worse, Don’t know

Independent variables

*Overall satisfaction with housing organisation (Source: Scottish Social Housing Charter indicators)*

Overall, how satisfied are you with [participant organisation]?

Options: Very satisfied, Satisfied, Neither, Unsatisfied, Very unsatisfied, Don’t know

*Comparison of current and previous experience of renting*

Would you say your experience of renting this property is... than your previous experience of renting?

Options: A lot better, A little better, The same, A little worse, A lot worse, Don’t know, N/A

*Rating of property quality (Source: Scottish House Condition Survey – part of Scottish Household Survey)*

Overall, would you say the general condition of the property you're currently living in is...

Options: Very good, Good, Satisfactory, Poor, Very poor, Don’t know

*Satisfaction with maintenance service (Source: Scottish House Condition Survey – part of Scottish Household Survey)*

Overall, how satisfied are you with the quality of the maintenance you currently get?

Options: Very satisfied, Satisfied, Neither, Unsatisfied, Very unsatisfied, Don’t know

*Rating of ability to cope financially over the last few months (Source: Scottish Household Survey – amended)*

Overall, over the last few months, would you say it’s been easy to make ends meet...

Options: All of the time, Most of the time, Some of the time, Hardly ever, Never, Don’t know

*Rating of ability to cope with paying rent over the last few months (Source: Scottish Household Survey – amended)*

Thinking specifically about paying your rent, would you say you generally feel able to cope with

paying your rent...

Options: All of the time, Most of the time, Some of the time, Hardly ever, Never, Don’t know

*Neighbourhood quality (Source: Scottish Household Survey)*

How would you rate your current neighbourhood as a place to live?

Options: Very good, Fairly good, Poor, Very poor, Don’t know

*Social support index (Source: Scottish Household Survey)*

How much do you agree or disagree with the following:

- People in this neighbourhood can be trusted
- I regularly stop and talk with people in my neighbourhood
- If I was alone and needed help, I could rely on one of my friends or relatives in this neighbourhood to help me
- The friendships and associations I have with other people in my neighbourhood mean a lot to me

Options: Strongly agree, Tend to agree, Neither agree not disagree, Tend to disagree, Strongly disagree, Don’t know

Responses coded 5-1 and mean used as index.
